# Type 1 Diabetes and Non-Alcoholic Fatty Liver Disease: When Should We Be Concerned? A Nationwide Study in Brazil †

**DOI:** 10.3390/nu9080878

**Published:** 2017-08-15

**Authors:** Bianca Senger Vasconcelos Barros, Deborah Conte Santos, Marcela Haas Pizarro, Laura Gomes Nunes de Melo, Marilia Brito Gomes

**Affiliations:** 1 Department of Internal Medicine, Diabetes Unit, State University of Rio de Janeiro (UERJ), Boulevard 28 de Setembro, 77-3º andar-Vila Isabel, Rio de Janeiro-RJ CEP 20551-030, Brazil; deborahconte@hotmail.com (D.C.S.); marcelahpizarro@gmail.com (M.H.P.); mariliabgomes@gmail.com (M.B.G.); 2 Department of Ophthalmology, State University of Rio de Janeiro (UERJ), Boulevard 28 de Setembro, 77-4º andar-Vila Isabel, Rio de Janeiro-RJ CEP 20551-030, Brazil; lauragnmelo@gmail.com

**Keywords:** type 1 diabetes, metabolic syndrome, aminotransferase, liver, NAFLD, obesity

## Abstract

Obesity is increasing worldwide, affecting even patients with type 1 diabetes (T1D). A higher prevalence of associated comorbidities is expected, such as non-alcoholic fatty liver disease (NAFLD). This paper reports a cross-sectional multicenter study on a population with T1D (*n* = 1662), which aimed to evaluate the prevalence of metabolic syndrome (MS), a known risk factor for NAFLD, and to investigate predisposing factors associated with MS, as well as factors associated with elevated alanine aminotransferase (ALT), as it correlates to liver fat content. Patients were from 14 public clinics of 10 cities from all geographical regions of Brazil. A high prevalence of MS was found, especially among adults (32.3%), and this was related to age, female gender, acid uric levels, and the presence of acanthosis nigricans. ALT above the normal range was associated with triglyceride levels (especially above 129.5 mg/dL), serum uric acid, age, male gender, HbA1c, and non-Caucasian ethnicity. Patients with T1D, metabolic syndrome, and the aforementioned factors may be at a higher risk of NAFLD and should be referred to ultrasound for NAFLD evaluation. Further studies are necessary to establish the prevalence of NAFLD in individuals with T1D and to determine the disease’s progression in these patients.

## 1. Introduction

Type 1 diabetes (T1D) is an increasingly recognized disease worldwide, associated with enhanced risk of micro- and macrovascular complications, comorbidities, reduced life expectancy, and higher health care costs [1,2,3,4,5]. One of the morbidities recently described in this disease is obesity [6,7], whose incidence is also increasing worldwide. The prevalence of obesity has doubled between 1980 and 2014, and it was estimated that in 2014 almost 2 billion adults were overweight or obese [8], according to the World Health Organization.

The obesity epidemic also affects the clinical features of T1D, usually diagnosed in a lean patient, with more cases being recognized in individuals with overweight and even obesity [9]. Furthermore, the intensive glycemic control associated with increased use of new insulin analogues and carbohydrate counting has led to weight gain in individuals already diagnosed with this disease [10]. Several studies have found that about 30% of patients with T1D are overweight or obese during follow-up in tertiary hospitals [11,12,13,14]. With these changes, recent findings exhibit the trend of type 1 diabetic patients with an intermediate profile, mixing characteristics previously considered “typical” of type 2 diabetes (T2D), such as high body mass index (BMI), increased abdominal circumference, and insulin resistance. Some authors have referred to these patients as having “double diabetes”, with the risk of all the complications associated with metabolic syndrome (MS) [6,15,16,17], and thus a higher susceptibility to non-alcoholic fatty liver disease (NAFLD).

NAFLD is the most common cause of chronic liver disease, and affects 25 to 30% of the world’s population. It is defined as the presence of ≥5–10% of hepatocytes with fat deposits, ranging from steatosis to high degrees of inflammation. NAFLD is often diagnosed in asymptomatic individuals without excessive alcohol intake (<20 g/day for women and <30 g/day for men), with persistent increase in aminotransferases, particularly alanine aminotransferase (ALT) or those with normal aminotransferases but with metabolic syndrome. These patients are usually referred to abdominal ultrasound. The finding of increased hepatic echogenicity leads to the clinical diagnosis of the disease, although definitive diagnosis depends on liver biopsy, performed only in specific situations, since it is an invasive procedure that is not required for the treatment this condition. NAFLD increases the risk of cardiovascular disease and hinders the achievement of glycemic goals, since it is associated with insulin resistance [18,19,20]. 

Due to these changes in the clinical and metabolic profile of T1D, we aimed to evaluate clinical and laboratory parameters that can be used as initial screening tests for fatty liver disease in T1D, as most studies approach only T2D. We also investigated the prevalence of metabolic syndrome in a multicenter population of T1D patients in Brazil and its relationship with elevated aminotransferases.

## 2. Materials and Methods

This was a cross-sectional study, based in a multicenter cohort of T1D patients, evaluated between August 2011 and August 2014 at 14 public clinics of secondary and tertiary care, located in 10 cities in all Brazilian geographic regions (North/Northeast, Midwest, Southeast, and South). The methods have been described previously [21]. 

Briefly, all patients attended secondary and tertiary clinics from the National Brazilian Health Care System and were seen by an endocrinologist. They had access to free neutral protamine Hagedorn (NPH) and regular insulin, syringes, needles, glucometers, and strips for blood glucose monitoring.

The inclusion criteria were: patients were older than or equal to 13 years of age, diagnosed by a physician with T1D through classical clinical findings (hyperglycemia, polyuria, weight loss, polydipsia, polyphagia, and the need for insulin therapy since diagnosis), and had attended medical follow-up for at least six months in each center.

The exclusion criteria included: being pregnant or breastfeeding at the time of inclusion; acute infectious process or history of ketoacidosis in the three months prior to recruitment; or exacerbation of comorbidities, such as congestive heart failure, cardiac arrhythmias, acute respiratory failure, or severe obstructive lung disease at the time of enrollment.

Each center provided data for at least 50 T1D patients, which were analyzed by a trained coordinator. Participants underwent a clinical-demographic survey by a standardized questionnaire in which data were collected on gender, current age, self-reported ethnicity, age at diagnosis, as well as diabetes duration, diet (characteristics and adhesion in last month), level of physical activity, smoking (defined as the current use of more than one cigarette per day), alcohol consumption, type of insulin and daily dose, frequency of self-monitoring of blood glucose, self-reported frequency of hypoglycemia in the last month (number of times the patient had blood glucose levels ≤70 mg/dL or needed help to overcome hypoglycemic symptoms), use of other medications, associated diseases, and hospitalization due to any cause in the last year. Economic status was evaluated according to the Brazilian Economic Classification Criteria, which also considers education status. 

Participants were examined and the following clinical variables were evaluated in medical visits: weight (kilograms), height (centimeters), BMI, blood pressure (BP), heart rate (HR), waist circumference (WC; determined at half the distance between the last costal arch and the iliac crest), and the presence of acanthosis nigricans.

The following laboratory variables were obtained at the last clinical visit: fasting plasma glucose, glycated hemoglobin A1c (high performance liquid chromatography; reference values: 4.0–6.0%), urea, creatinine, total cholesterol (TC), HDL cholesterol (HDL-c), triglycerides (TG) and LDL cholesterol (LDL-c) calculated by Friedewald’s equation (LDL-c = TC-TG/5-HDL-c, unless TG > 400 mg/dL), serum aminotransferases (AST and ALT), ultrasensitive C-reactive protein (CRP), complete blood count, and speed erythrocyte sedimentation. Fasting plasma glucose, TG, HDL-c, and TC were measured using enzymatic techniques.

Our original sample included 1760 patients. All patients were diagnosed with T1D between 1960 and 2014. Patients between 13 and 18 years of age were classified as adolescents, and patients 19 years of age or older were considered adults, based on the American Diabetes Association (ADA) criteria.

Of the 1760 patients, we subsequently excluded those with known liver disease, including viral hepatitis, hemochromatosis, alcoholic fatty liver disease, autoimmune hepatitis, cirrhosis, and hepatocellular carcinoma, as well as those who were using medications that are known to cause NAFLD: corticosteroids, methotrexate, amiodarone, and tamoxifen. Twelve individuals using corticosteroids and five with known liver disease were excluded. Also, we excluded 45 patients without data on aminotransferases and 36 patients with missing data on metabolic syndrome criteria.

The study was approved by the Ethics Committee of Pedro Ernesto University Hospital, at State University of Rio de Janeiro (protocol: CEP/HUPE 2769/2010), which was the coordinator center, and by the local ethics committee of each center. All participants or their guardians signed informed consent. Investigations were carried out following the rules of the Declaration of Helsinki.

### 2.1. Evaluation of Metabolic Syndrome and Aminotransferases

Patients were stratified according to metabolic syndrome criteria by the International Diabetes Federation (IDF) [22]. Considering they all have diabetes, patients that had the obligatory criterion of central obesity plus one other factor were classified as having metabolic syndrome, completing three positive criteria. The criteria used for the adults were the following: (1) abdominal obesity: WC ≥ 90 cm in South American men, ≥80 cm in South American women; (2) TG ≥ 150 mg/dL (1.7 mmol/L) or on drug therapy for elevated triglycerides; (3) HDL-c < 40 mg/dL (1.03 mmol/L) in men or <50 mg/dL (1.29 mmol/L) in women or on drug therapy for low HDL-c; (4) elevated BP ≥ 130 × 85 mmHg or receiving antihypertensive medications. 

Adolescents aged 16 years or older were classified according to the same criteria as adults. Adolescents aged 13 to 15 years old were classified according to the following criteria: (1) abdominal obesity: WC > 90th percentile for age and sex; (2) TG ≥ 150 mg/dL (1.7 mmol/L); (3) HDL-c < 40 mg/dL (1.03 mmol/L); (4) elevated BP ≥ 130 × 85 mmHg [23]. As there are no reference values of percentiles on abdominal waist in the Brazilian population, we used the 90th percentile for each age and gender group of our sample. 

Further analysis was performed with patients stratified into two groups: normal vs. elevated aminotransferases, according to the range of normality recommended by the American College of Gastroenterology (ALT ≤ 25 U/L for women and ≤ 33 U/L for men) [24]. Then, we investigated which clinical and laboratory parameters were associated with elevated ALT.

### 2.2. Statistical Analysis 

Continuous variables are expressed as means ± standard deviations (SD) or medians [interquartile range], and categorical variables as frequencies and percentages. For the analysis of the differences of categorical variables, the Chi-square or Fisher’s exact test was used, when indicated. To compare the means of the independent continuous variables, Student’ *t*-tests or Mann-Whitney U test was used. A linear-by-linear association was performed to evaluate the relationship between quartiles of aminotransferases and the prevalence of metabolic syndrome. A binary logistic regression was performed to access the predisposing factors to metabolic syndrome, considering clinically relevant factors that were statistically significant in the descriptive analysis and that were not involved in the diagnostic criteria. The following factors were included: gender, age, diabetes duration (years), uric acid levels, family history of type 2 diabetes and family history of obesity in first degree relatives, presence of acanthosis nigricans, years of education, and practice of physical exercise. The Nagelkerke *R*^2^ value was calculated. Odds ratios with 95% confidence intervals (CI) are expressed as indicated. In another analysis, a stepwise linear regression was performed to determine factors that influence ALT levels. The same factors used on the binary logistic regression were included in addition to the criteria of metabolic syndrome, use of medications (metformin, fibrate, statins), uric acid levels, HbA1c, and self-reported ethnicity. A receiver operating characteristics (ROC) curve was also performed in an attempt to establish the best cut-off for triglycerides that discriminates elevated ALT in this population with T1D. Differences were considered significant at a two-sided *p*-value less than 0.05. All statistical analyses were performed with Statistical Package for Social Sciences (SPSS) 24.0 (SPSS, Chicago, IL, USA).

## 3. Results

### 3.1. Baseline Characteristics and Prevalence of Metabolic Syndrome

A total of 1662 individuals were analyzed: 55.2% were female, with a mean age of 30 ± 12 years, 53.9% were Caucasian, from the low (47%) or middle (43.7%) social class. Diabetes duration was 15.5 ± 9.3 years for the whole population study, with a significant difference between adults and teenagers (17.1 ± 9.3 years vs. 7.7 ± 4.3 years, *p* < 0.01).

According to the IDF, 32.3% of the adults and 8.4% of the adolescents were classified as having metabolic syndrome. Data are shown in Table 1. As expected, adults with MS had a higher prevalence of other comorbidities such as hypertension, dyslipidemia, higher prevalence of overweight and obesity, and use of medication, in comparison to those without MS. They also had higher levels of aminotransferases, more alterations in serum lipids parameters, higher levels of C-reactive protein and uric acid, and lower glomerular filtration rate. Adults with MS exhibited a higher prevalence of family history of T2D and obesity, higher prevalence of acanthosis nigricans, and lower practice of physical exercise. Adolescents with MS showed similar differences regarding lower glomerular filtration rate, lower HDL-c, higher blood pressure, WC, BMI, and higher use of statin and metformin. The others parameters were not altered, as in adults. Glycated hemoglobin and diet adherence were not significant in any analysis. There were no differences in the mean of the years of education or in the geographic region distribution.

### 3.2. Evaluation of Alanine Aminotransferase Levels

A linear-by-linear association was observed between quartiles of ALT levels and the prevalence of MS (*p* < 0.001).

A total of 110 patients had ALT above the range of normality recommended by the American College of Gastroenterology (ALT > 25 U/L for women and >33 U/L for men) [24], 48 of which belonged to the group with MS. This corresponds to 10.2% of patients with MS versus 5.2% without MS (*p* < 0.001). 

In the logistic regression analysis, 99.2% of patients were included. The model was statistically significant, χ^2^ = 280.723, *p* < 0.001, and explained 22.5% (Nagelkerke *R*^2^) of the variance in metabolic syndrome. The odds of having MS was 2.63 for females compared to males (CI 95% 2.02–3.42; *p* < 0.001). Acanthosis nigricans exhibited the highest odds (OR: 5.33 CI 95% 2.97–9.58; *p* < 0.001), and age and acid uric levels were also significant predisposing factors to MS. Data are shown in Table 2. 

A linear regression established that ALT levels have a direct relationship with triglyceride levels, serum uric acid, self-reported ethnicity as non-Caucasian, age, male gender, and HbA1c. A total of 95.7% of patients were included in this analysis. Data are shown in Table 3.

**Figure 1:**
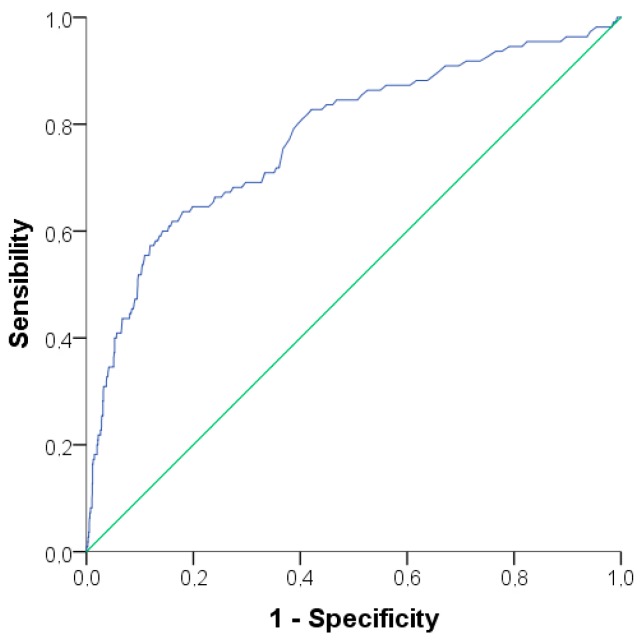
Receiver Operating Characteristics curve for predicting elevated alanine transaminase levels (>25 UI/L in women and >33 UI/L in men) with triglyceride values. Area under the curve: 0.777 (CI 95% 0.726–0.827; *p* < 0.001). Triglycerides levels of 129.5 mg/dL provide a sensitivity of 64.5% and specificity of 80.2%.

In the ROC curve analysis (Figure 1), the area under the curve was 0.777 (CI 95% 0.726–0.827; *p* < 0.001) and a triglycerides level of 129.5 mg/dL established the highest acceptable sensitivity of 64.5% and specificity of 80.2% to detect high levels of ALT. 

## 4. Discussion

The present study showed that patients with type 1 diabetes in Brazil have a high prevalence of metabolic syndrome. Adults with MS had higher rates of comorbidities such as hypertension and dyslipidemia, as well as parameters linked to insulin resistance such as higher serum acid uric, higher rates of acanthosis nigricans, and a more frequent use of metformin. Adolescents did not show as many alterations, probably due to shorter diabetes duration. Although we found significantly lower doses of daily insulin in the group with MS compared to the group without MS, HbA1c levels were similar in these groups and the small difference does not seem to have a clinical impact. Diet adherence was not related to the presence of MS. However, this might be related to information bias, since it was self-reported. Even so, diet is still recognized as an important treatment tool for metabolic syndrome and should always be reinforced. Predisposing factors to MS were female gender, age, acid uric levels, and the presence of acanthosis nigricans. Both groups, with and without metabolic syndrome, had poor glycemic control, as shown by HbA1c and FPG levels. Good glycemic control is still an important issue in Brazil, related to insulin and diet adherence. Even among patients with good adherence, the majority does not have HbA1c in their target range [12,21]. We cannot predict if the results would be the same if patients had adequate glycemic control.

Because MS is a known risk factor for NAFLD, most guidelines suggest that these individuals have their levels of aminotransferases evaluated, especially ALT, as it correlates to liver fat content [18,20,24]. If ALT levels are above reference values, further investigation is recommended, usually with ultrasound. In our study, a prevalence of 10% of individuals with elevated ALT was observed. As previous studies on ALT levels in T1D population are not available, we cannot establish the magnitude of this finding. However, even among patients with normal ALT, these levels were significantly higher in the group with MS. 

Regarding the criteria for metabolic syndrome, triglyceride levels were the most relevant parameter in the linear regression. They showed a direct association with ALT levels, even after correction for the use of lipid-lowering drugs. For each 10 mg/dL increase in triglyceride levels, ALT increases 0.5 UI/L. Serum uric acid, age, male gender, and HbA1c also showed a positive relation with ALT increase. Uric acid is a product of fructose metabolism [25] in the liver, and has been demonstrated to be associated with systemic inflammation, insulin resistance, and NAFLD [26,27,28,29]. Therefore, increase in ALT caused by uric acid may be related to fatty liver damage.

Particular strengths of our study include the multicenter design with standardized protocol, the large admixed population sample of type 1 diabetic patients, and the study population from across all geographic regions of Brazil. 

Our study has some limitations. First, there are different classifications for MS. IDF classification was chosen because it establishes different reference values of waist circumference according to ethnicity. As we have analyzed a highly mixed population, this seemed to be the best option. Also, in the classification for MS, the use of angiotensin converter enzyme inhibitor or angiotensin 2 receptor blocker was not considered for the hypertension criterion, as it is frequently used in patients with albuminuria. Mean blood pressure and/or diagnosis of hypertension recorded on medical charts were used. Still, we found a high prevalence of metabolic syndrome, especially in adults. Second, a misclassification of diabetic patients could have occurred because we did not consider autoantibodies or C-peptide levels for the diagnosis of T1D. However, we do not believe that this has great impact in our results, since 93.2% of the studied population was diagnosed before 30 years old, and most likely were correctly classified. Third, liver images and biopsies for the diagnosis of NAFLD were not performed because in this study we did not intended to diagnose NAFLD, but to established the possible predictors for screening. 

NAFLD is an important cause of liver disease, associated with cardiovascular disease, cirrhosis, liver cancer, and impaired glucose control. Most studies approach only type 2 diabetes, because of its association with hepatic insulin resistance that leads to an inability to suppress hepatic glucose production and in turn, favors fatty acid liver deposits. Although type 1 diabetic patients have low levels of hepatic insulin in comparison to type 2 diabetics, thereby favoring β oxidation, it is still controversial whether this protects [30] them against NAFLD. Some studies have associated T1D to insulin resistance and NAFLD [31,32], while a recent study [33] showed that NAFLD was related to BMI, age, gender, and the use of statins, but not to the presence of T1D, although the number of cases in this study was very small (nine patients with T1D and NAFLD). Nonetheless, in times of high prevalence of obesity worldwide, this complication deserves attention even in the type 1 diabetic population.

Although reference values for ALT have frequently been questioned by specialists [24] and standardized values for the Brazilian population are not available, patients with T1D and metabolic syndrome have higher ALT levels that might be related to hepatic lesion, especially if they are associated with non-Caucasian ethnicity, male gender, elevated triglycerides (>129.5 mg/dL), uric acid, and HbA1c. Therefore, those patients should be referred to NAFLD screening, initially with ultrasound, since it is a non-invasive and low-cost exam.

## 5. Conclusions

Patients with T1D and metabolic syndrome may be at a high risk of NAFLD. More studies are necessary to establish the prevalence of NAFLD and to define the progression of this disease in type 1 diabetic patients.

## Figures and Tables

**Table 1:** Comparison of clinical and laboratorial parameters between groups with and without metabolic syndrome, stratified by age groups.

	Adults			Adolescents		
	MS^+^	MS^−^	*p*-Value	MS^+^	MS^−^	*p*-Value
*N* (%)	445 (32.3)	931 (67.7)		24 (8.4)	262 (91.6)	
**Clinical parameters**						
Age, years	36.3 ± 11.5	31.4 ± 10.5	<0.001	16.9 ± 1.3	15.7 ± 1.7	<0.001
Female gender, *n* (%)	304 (68.3)	478 (51.3)	<0.001	18 (75.0)	118 (45.0)	0.005
Diabetes duration, years	19.2 ± 9.7	16.0 ± 8.9	<0.001	9.1 ± 4.9	7.6 ± 4.2	0.11
Years of education	12.2 ± 4.3	12.7 ± 3.8	0.02	11.0 ± 2.8	10.4 ± 2.3	0.27
WC (centimeters)	94.2 ± 9.6	79.1 ± 9.0	<0.001	89.4 ± 7.1	75.3 ± 8.3	<0.001
BMI (kg/m^2^)	27.8 ± 4.2	23.1 ± 3.2	<0.001	27.0 ± 3.4	21.3 ± 3.0	<0.001
SBP (mmHg)	129.4 ± 16.8	120.9 ± 15.1	<0.001	120.3 ± 10.9	112.2 ± 11.7	0.002
DBP (mmHg)	79.7 ± 10.1	74.5 ± 9.7	<0.001	76.3 ± 8.3	69.1 ± 9.1	<0.001
Hypertension, *n* (%)	175 (39.3)	117 (12.6)	<0.001	0	1 (0.4)	1.0
Dyslipidemia, *n* (%)	149 (33.5)	182 (19.5)	<0.001	3 (12.5)	21 (8.0)	0.44
Obesity, *n* (%)	98 (22.0)	30 (3.2)	<0.001	5 (20.8)	8 (3.1)	<0.001
Overweight, *n* (%)	228 (51.2)	189 (20.3)	<0.001	10 (41.7)	36 (13.7)	<0.001
Smoking, *n* (%)	21 (4.7)	55 (5.9)	0.37	1 (4.2)	11 (4.2)	1.0
Metformin use, *n* (%)	105 (23.1)	70 (7.5)	<0.001	12 (50.0)	15 (5.7)	<0.001
Statin use, *n* (%)	155 (34.0)	194 (20.8)	<0.001	4 (16.7)	6 (2.3)	0.006
Fibrate use, *n* (%)	8 (1.8)	5 (0.5)	0.03	-	-	-
ACEi or AT2 blocker use, *n* (%)	201 (44.1)	226 (24.3)	<0.001	2 (8.3)	19 (7.3)	0.68
Total daily insulin (U/kg)	0.8 ± 0.3	0.9 ± 0.4	0.02	0.9 ± 0.4	1.1 ± 0.4	0.048
Family history of T2D, *n* (%)	151 (33.9)	232 (24.9)	0.001	5 (20.8)	28 (10.7)	0.172
Family history of obesity, *n* (%)	130 (28.5)	196 (21.1)	0.001	6 (25.0)	58 (22.1)	0.798
Diet adherence ≥ 80%, *n* (%)	212 (47.6)	510 (54.7)	0.059	10 (50.0)	119 (50.9)	1.0
Physical exercise, yes *n* (%)	192 (43.1)	477 (51.2)	0.004	13 (54.2)	174 (66.4)	0.264
Acanthosis nigricans, *n* (%)	46 (10.3)	15 (1.6)	<0.001	3 (12.5)	4 (1.5)	0.015
**Laboratorial parameters**						
ALT (U/L)	16.2 ± 12.6	13.1 ± 9.3	<0.001	13.0 ± 10.0	12.8 ± 9.4	0.94
AST (U/L)	23.7 ± 19.5	19.4 ± 12.2	<0.001	17.3 ± 14.3	19.8 ± 12.8	0.38
Albumin (mg/dL)	3.9 ± 0.7	3.9 ± 0.5	0.62	3.9 ± 0.5	4.0 ± 0.6	0.21
GGT (U/L)	21 (21)	18 (13)	<0.001	19 (17.5)	16 (9)	0.41
FPG (mg/dL)	171.0 (132.5)	166.5 (155.2)	0.99	152.5 (166.7)	200.0 (161.7)	0.13
HbA1c (%)	8.9 ± 1.9	8.8 ± 2.1	0.96	9.5 ± 2.5	9.7 ± 2.4	0.73
HbA1c (mmol)	73.3 ± 20.7	73.3 ± 22.8	0.96	80.9 ± 27.9	82.8 ± 26.6	0.73
RCP (mg/dL)	0.7 ± 1.5	0.4 ± 1.0	<0.001	0.4 ± 0.5	0.3 ± 0.6	0.17
GFR (ml/min)	75.5 ± 25.6	84.0 ± 25.8	<0.001	102.5 ± 26.2	115.7 ± 30.8	0.03
TC (mg/dL)	198.8 ± 64.3	184.2 ± 41.4	<0.001	184.6 ± 55.5	185.3 ± 57.1	0.95
LDL-c (mg/dL)	117.3 ± 47.1	107.2 ± 34.5	<0.001	111.7 ± 41.0	106.7 ± 37.3	0.54
HDL-c (mg/dL)	53.8 ± 20.9	58.1 ± 17.6	<0.001	46.3 ± 13.0	55.7 ± 16.6	0.007
Triglycerides (mg/dL)	80 (95.5)	107 (51)	<0.001	88.5 (131)	84 (59)	0.15
Uric acid (mg/dL)	5.6 ± 2.1	4.9 ± 1.7	<0.001	5.0 ± 1.8	4.9 ± 1.4	0.78

Data are represented as means ± standard deviation, median [interquartile range], or as numbers (percentages). *p*-value refers to the comparison between the group with (MS^+^) and without (MS^−^) metabolic syndrome. WC: waist circumference; BMI: body mass index; SBP: systolic blood pressure; DBP: diastolic blood pressure; ACEi: angiotensin converter enzyme inhibitor; AT2 blocker: Angiotensin 2 receptor blocker; T2D: type 2 diabetes; ALT: alanine aminotransferase; AST: aspartate aminotransferase; GGT: gamma-glutamyl transferase; FPG: fasting plasma glucose; HbA1c: glycated hemoglobin; RCP: reactive C protein; GFR: glomerular filtration rate; TC: total cholesterol; LDL-c: low density lipoprotein cholesterol; HDL-c: high density lipoprotein cholesterol. Family history refers to first degree relatives.

**Table 2:** Predisposing factors to metabolic syndrome, accessed by binary logistic regression.

Variable	B	OR	95% CI	*p*-Value
Age (years)	0.044	1.04	1.03–1.06	<0.001
Female gender	0.967	2.63	2.02–3.42	<0.001
Years of education	0.010	1.01	0.98–1.04	0.531
Diabetes duration (years)	0.007	1.01	0.99–1.02	0.364
Acid uric levels (mg/dL)	0.265	1.30	1.22–1.40	<0.001
Practice of physical exercise, yes	−0.171	0.84	0.66–1.07	0.165
Presence of acanthosis nigricans, yes	0.1674	5.33	2.97–9.58	<0.001
Positive family history of T2D	0.044	0.75	0.79–1.38	0.752
Positive family history of obesity	0.195	1.22	0.92–1.60	0.164

B: coefficient for logistic regression; OR: odds ratio; 95% CI: 95% confidence interval.

**Table 3:** Linear regression for predicting ALT levels.

Model	*R*	Adjusted *R*^2^	Variables	B	95% CI for B	*p*-Value
1	0.437	0.191	triglycerides (mg/dL)	0.06	0.05–0.06	<0.001
2	0.446	0.199	triglycerides (mg/dL)	0.05	0.04–0.06	<0.001
			uric acid (mg/dL)	0.55	0.27–0.83	<0.001
3	0.452	0.202	triglycerides (mg/dL)	0.05	0.04–0.06	<0.001
			uric acid (mg/dL)	0.54	0.26–0.81	<0.001
			non-Caucasian ethnicity	1.52	0.61–2.44	0.001
4	0.456	0.206	triglycerides (mg/dL)	0.05	0.04–0.06	<0.001
			uric acid (mg/dL)	0.50	0.22–0.78	<0.001
			non-Caucasian ethnicity	1.68	0.76–2.60	<0.001
			age (years)	0.06	0.02–0.09	0.004
5	0.461	0.210	triglycerides (mg/dL)	0.05	0.05–0.06	<0.001
			uric acid (mg/dL)	0.38	0.09–0.67	0.009
			non-Caucasian ethnicity	1.66	0.75–2.58	<0.001
			age (years)	0.06	0.02–0.09	0.002
			male gender	1.50	0.55–2.44	0.002
6	0.464	0.212	triglycerides (mg/dL)	0.05	0.04–0.06	<0.001
			uric acid (mg/dL)	0.40	0.11–0.69	0.007
			non-Caucasian ethnicity	1.54	0.62–2.46	0.001
			age (years)	0.07	0.03–0.11	0.001
			male gender	1.55	0.60–2.50	0.001
			HbA1c (%)	0.26	0.03–0.48	0.023

ALT: alanine aminotransferase; HbA1c: glycated hemoglobin.
